# An Open-Source Deep Learning-Based GUI Toolbox for Automated Auditory Brainstem Response Analyses (ABRA)

**DOI:** 10.21203/rs.3.rs-6735294/v1

**Published:** 2025-06-20

**Authors:** Abhijeeth Erra, Jeffrey Chen, Cayla M. Miller, Elena Chrysostomou, Shannon Barret, Yasmin M. Kassim, Rick A. Friedman, Amanda Lauer, Federico Ceriani, Walter Marcotti, Cody Carroll, Uri Manor

**Affiliations:** 1 Data Institute, University of San Francisco, San Francisco, CA; 2 Dept. of Cell & Developmental Biology, University of California San Diego, La Jolla, CA,; 3 Dept. of Otolaryngology, University of California San Diego, La Jolla, CA; 4 Depts. of Otolaryngology-Head and Neck Surgery and Neuroscience and Center for Functional Anatomy and Evolution, Johns Hopkins University School of Medicine, Baltimore, MD; 5 School of Biosciences, University of Sheffield, Sheffield, S10 2TN, UK.; 6 Neuroscience Institute, University of Sheffield, Sheffield, S10 2TN, UK; 7 Dept. of Mathematics and Statistics, University of San Francisco, San Francisco, CA; 8 Halıcıoğlu Data Science Institute, University of California San Diego, La Jolla, CA

## Abstract

Hearing loss is a pervasive global health challenge with profound impacts on communication, cognitive function, and quality of life. Recent studies have established age-related hearing loss as a significant risk factor for dementia, highlighting the importance of hearing loss research. Auditory brainstem responses (ABRs), which are electrophysiological recordings of synchronized neural activity from the auditory nerve and brainstem, serve as in vivo readouts for sensory hair cell, synaptic integrity, hearing sensitivity, and other key features of auditory pathway functionality, making them highly valuable for both basic neuroscience research and clinical diagnostics. Despite their utility, traditional ABR analyses rely heavily on subjective manual interpretation, leading to considerable variability and limiting reproducibility across studies. Here, we introduce Auditory Brainstem Response Analyzer (ABRA), a novel open-source graphical user interface powered by deep learning, which automates and standardizes ABR waveform analysis. ABRA employs convolutional neural networks trained on diverse datasets collected from multiple experimental settings, achieving rapid and unbiased extraction of key ABR metrics, including peak amplitude, latency, and auditory threshold estimates. We demonstrate that ABRA’s deep learning models provide performance comparable to expert human annotators while dramatically reducing analysis time and enhancing reproducibility across datasets from different laboratories. By bridging hearing research, sensory neuroscience, and advanced computational techniques, ABRA facilitates broader interdisciplinary insights into auditory function. An online version of the tool is available for use at no cost at https://abra.ucsd.edu.

## Introduction

Hearing loss is a prevalent and debilitating condition affecting hundreds of millions worldwide. In addition to significantly diminishing quality of life, age-related hearing loss has emerged as a major risk factor for cognitive decline and dementia, underscoring the urgent need for better understanding and treatment strategies. Both age-related hearing loss and dementia involve the progressive loss of synapses (synaptopathy) — in the cochlea and brain, respectively — highlighting common neurodegenerative mechanisms that warrant intensive study (O’Leary et al. 2017; Bramhall et al. 2018; Loughrey et al. 2018; Ray et al. 2018; Liu et al. 2020; Livingston et al. 2020; Livingston et al. 2024; Park et al 2024). Mouse models have become indispensable in hearing research, owing to their genetic tractability and close anatomical similarity to human auditory biology.

**Figure 1:**
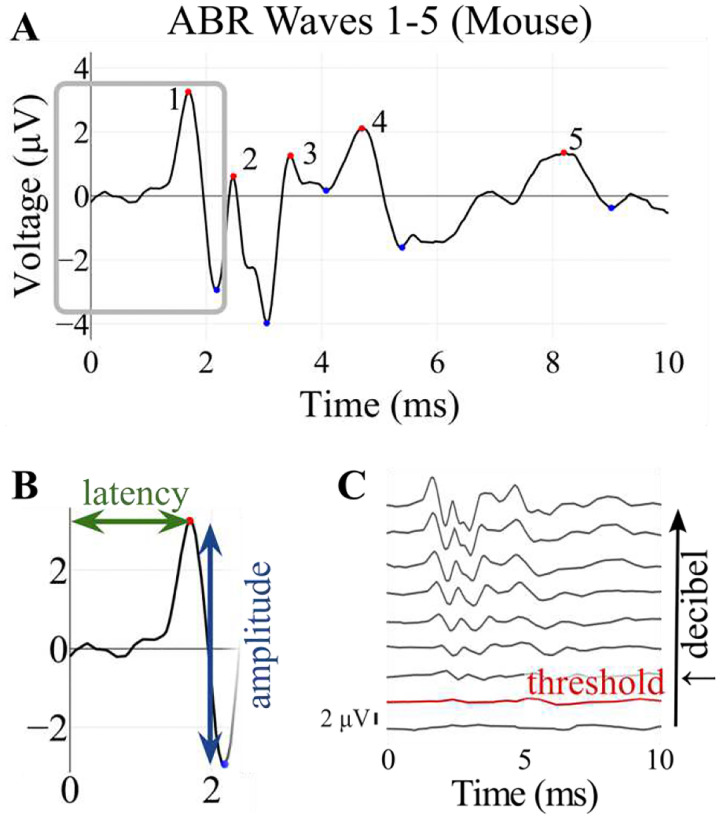
Example of ABR waveforms recorded from a mouse, showing its characteristic features. (A) One 10 ms ABR recording, with the five characteristic peaks denoted by red dots, and their corresponding troughs with blue dots. (B) A close up of the boxed region in (A), showing how latency (time to peak) and amplitude (peak to trough height) are defined for wave 1. (C) Several ABR recordings at varying decibels, for the same mouse and frequency. The threshold level above which the ABR response is indicative of hearing is shown in red.

Among the most powerful approaches to assess auditory function are auditory brainstem response (ABR) recordings, which objectively measure electrical activity along the auditory neural pathway, from cochlear inner hair cells through the brainstem (Eggermont 2019; Kim et al. 2022; Burkard and Don 2012; Ingham et al. 2011; Møller and Jannetta 1985; Xie et al. 2018). In mice, ABRs consist of five characteristic peaks approximately corresponding to neural signals propagating through sequential auditory structures, though some centrally generated waves may reflect concurrent activity in multiple structures (Figure 1, Rüttiger et al. 2017; Melcher et al. 1996; Henry 1979; Land et al. 2016).

Two particularly informative measurements obtained from ABRs—hearing threshold sensitivity and wave 1 characteristics—have been shown to correlate strongly with cochlear-based hearing impairment; specifically, hearing thresholds increase and wave 1 amplitudes are dampened if hair cells are damaged. (Fernandez et al., 2015; Bramhall et al. 2018; Bao et al. 2022; Young, Cornejo, and Spinner 2023). However, current ABR analysis methods typically rely on manual waveform interpretation, which can be subjective, labor-intensive, and prone to inconsistency between individual researchers or labs (Suthakar and Liberman 2019, Schrode 2022).

Heuristic and machine learning computational approaches have been explored for automated ABR analysis (Shaheen et al. 2024). Supervised learning models (i.e. models which learn from data with ground truth labels) like convolutional neural networks (CNNs), gradient boosting machines, and others have been used to accurately analyze suprathreshold ABR waveforms (Wimalarathna et al. 2021, McKearney and MacKinnon 2019, Kamerer et al. 2020) and to assess the degree of synaptopathy in humans (Buran et al. 2022).

In this paper, we introduce the Auditory Brainstem Response Analyzer (ABRA), a novel open-source software that implements a collection of machine learning models trained on a diverse range of mouse ABR datasets from multiple experimental settings for comprehensive and maximally generalizable mouse ABR analysis. ABRA is a user-friendly, browser-based application that supports batch data import/export, waveform visualization, automated peak detection, threshold estimation, latency quantification, time warping for curve alignment, and interactive 2D/3D plotting. By integrating these diverse functionalities into a unified platform, ABRA aims to streamline ABR data processing and analysis, reduce manual labor, and facilitate standardization and reproducibility across labs. We demonstrate ABRA’s flexibility and generalizability by benchmarking its performance on ABR datasets collected from three different hearing research labs using distinct experimental protocols and recording settings.

## Methods

### Data Collection

To test the generalizability and flexibility of developed open-source ABR software, we used three distinct datasets from different laboratories to train and evaluate ABRA’s models (Table 1). While the three laboratories used a similar overarching methodology, including anesthesia, electrode placement, and decibel (dB) ranges, each used unique experimental protocols, including varying collection software, sound source, and mouse strains. These differences underscore the flexibility of ABRA in accommodating diverse experimental setups and protocols. Further details on data collection conditions are available in the Supplementary Information (Supplementary Table S1).

### The ABRA Graphical User Interface

The proposed Auditory Brainstem Response Analyzer (ABRA) software was built to facilitate the examination and analysis of ABR waveforms. ABRA was developed in Python using the Streamlit framework (“Streamlit” n.d.) and provides an interactive platform for researchers to visualize ABR data. All documentation of the code for the GUI and instructions for using ABRA can be found at: https://doi.org/10.5281/zenodo.15054979 (Erra et al. 2025). ABRA allows users to import multiple ABR data files, and accepts data in most commonly used formats: in .arf or exported .csv formats for data collected with the BioSigRZ software (Tucker Davis Technologies) and in .asc or .tsv format for data collected with the CFTS software (Eaton-Peabody Laboratories). For other data types, ABRA will accept any data converted into a generalized .csv format, and an example template is provided. Upon import, the data is preprocessed to extract the timescale of the recording, the frequencies, dB levels, and the waveform data.

**Figure 2:**
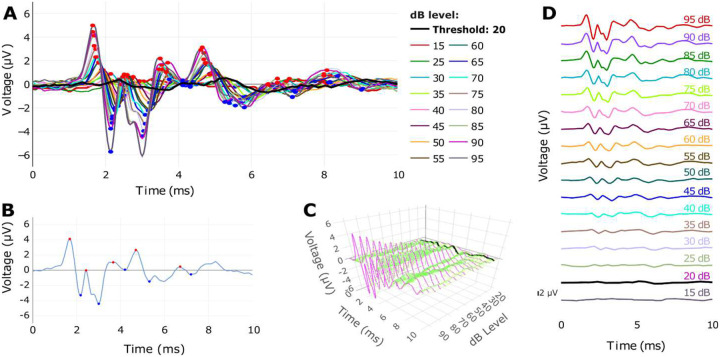
Visualizations from the ABRA app highlighting the various plotting functionalities on the same data. (A) visualizing several ABR waveforms from one 1 mo male C57Bl/6N mouse across different togglable dBs at 18 kHz with predicted peak locations (red points) and predicted threshold (thick black line). (B) plotting a single ABR waveform at a specific sound frequency (18 kHz) and intensity (85 dB) with peaks and troughs labeled. (C) 3D plotting of the same ABR waveforms in (A), with the predicted threshold (20 dB) highlighted in black (can be interactively rotated in the app) (D) stacked plotting of the same ABR waveforms at a single frequency, across increasing dB, with the predicted threshold (20 dB) highlighted in black.

After import and preprocessing, ABRA allows the user to select which frequencies and decibel levels they wish to examine for plotting and analysis. The ABR plots are shown through the Plotly framework in Python and can be downloaded as .png and .pdf files (Plotly Technologies Inc. 2015). ABRA calculates and displays metrics under the plots related to the displayed waveforms, including wave 1 amplitude, latency to the first peak, and threshold (as defined in Figure 1). These metrics can be downloaded as a.csv file. ABRA also allows the user to view all the waveforms for a single frequency, highlights the automatically detected peaks and troughs, and automates thresholding (Figure 2).

ABRA also implements two novel visualization features for ABR waveforms of multiple dBs at a given stimulation frequency. First, ABRA provides the option to implement time warping, which visually aligns the peaks and troughs of multiple waveforms (see Supplementary Figure S1). This view does not change the underlying data, and does not affect wave amplitudes, but stretches and compresses the waveform to varying degrees along the time axis to enhance visualization. Second, the app provides an interactive 3D surface plot of waveforms which allows the user to view the series of ABR waveforms as a surface in the 3-dimensional space created by the time domain, the probed decibel levels, and the recorded ABR voltage. ABRA’s various functionalities can provide the user with tools to visually ascertain features like thresholds and peaks, and automated model predictions of those features in tabular form. ABRA also provides automated batch analyses for multiple data files.

### ABRA Peak Detection

**Figure 3:**
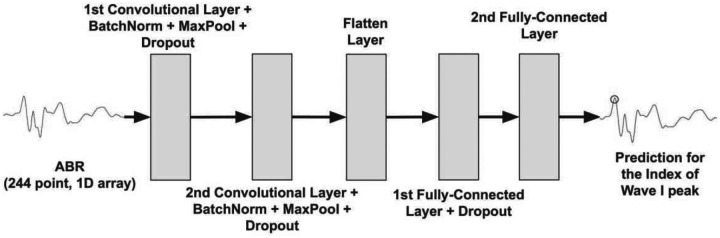
Model architecture for the wave 1 peak finding algorithm. The input (a 244-point ABR waveform spanning 10 ms) passes through two layers of convolution, batch normalization, max pooling (after ReLU activation), and dropout. The dimensionality of the output is then reduced through two consecutive fully-connected layers using ReLU activation which returns the prediction of the time point of the wave 1 peak.

ABRA incorporates a two-step peak finding algorithm that leverages Pytorch’s deep learning library and the Scikit-learn library. The first step involves deploying a Convolutional Neural Network (CNN) to retrieve a prediction for the location of the wave 1 peak. The CNN was trained on 7,209 ABRs (222 ABRs from 25 mice (Lab A); 6,987 ABRs from 67 mice (Lab B)) labeled with ground truth data related to the wave 1 peak and validated on 1,714 ABRs (66 ABRs from 7 mice (Lab A); 1,648 ABRs from 16 mice (Lab B)). Each training sample’s loss is weighted to ensure that each lab’s data is represented equally in the model training. Before training the CNN, each ABR waveform was rescaled as follows: a standardized scaler applied over the waveform generated z-scores for each timepoint, and a min-max scaler on those z-scores scaled each wave between 0 and 1. Each input ABR, recorded over 10 ms, was a vector of length 244. ABRs not initially sampled at 244 samples per 10 ms were truncated to 10 ms and either downsampled to 244 points using linear interpolation or upsampled to 244 points using cubic spline interpolation. The dataset was split into two sets with 80% of subjects from each lab going into the training set and 20% of subjects from each lab going into the testing set. Twenty percent (20%) of the training set subjects were used for validation. The CNN optimizes squared error loss (L^2^) for the regression task which returns a prediction for the wave 1 peak index. The validation set was evaluated at each training epoch, and if the validation loss did not decrease over 25 consecutive epochs, training was halted to prevent overfitting. With this model, training stopped after 62 epochs. A simplified representation of the network architecture is shown in Figure 3. Model hyperparameters chosen by cross-validation are displayed in Supplementary Table S2.

The CNN’s prediction of the wave 1 peak location serves as a reasonable initial estimate, but ABRA performs further fine-tuning in order to ensure that it is not sitting at a point neighboring the peak. First, the ABR was smoothed using Gaussian smoothing to attenuate or remove nuisance noise to identify peak indices. Then the *find_peaks* method from Scikit-learn was used to identify the remaining wave 2–5 peak/trough locations and voltages. This was done by searching for all local maxima and minima by simple comparison of neighboring values of the wave starting from the CNN predicted wave 1 peak index. Afterwards, the unsmoothed waveforms are utilized to quantify the amplitudes at the previously identified indices. The parameters for these methods were optimized using ground truth wave 1 latency and wave 1 amplitude for the validation set (1,714 ABRs). These parameters include the following:

- Window size for the start point for the smoothed waveform being inputted into the *find_peaks* function (optimized to 0.4098 ms before the CNN prediction for wave 1 peak).
- Time between peaks such that the correct peaks are identified (optimized to 0.6557 ms).
- Bandwidth parameter for the Gaussian smoothing step was set to *σ* = 1.0 to maximize alignment with human-labeled ground truth peaks.
- Time between troughs such that the correct troughs are identified (optimized to 0.2869 ms).

These choices of tuning parameters ensured that ABRA’s peak detection was robust across both high- and low-SNR conditions of ABRs in the training and testing data. Note that this approach may fail for certain conditions where latency is particularly abnormal.

### Supervised Threshold Estimation with ABRA

ABRA’s threshold estimation method uses a binary machine learning classifier to identify individual ABR waveforms as either above or below threshold, where above threshold is taken as the positive class. Once individual waveforms are classified, the hearing threshold for a given frequency is determined as the quietest stimulus level (in dB) for which a subject’s ABR waveform is classified as a hearing response (i.e. above threshold). Three candidate supervised binary classifiers were trained and evaluated: A CNN, an XGBoost classifier, and a Logistic Regression Classifier.

Evaluation metrics include accuracy, true positive rate (TPR; i.e. the proportion of actual above threshold instances that are correctly identified as above threshold), false positive rate (FPR; i.e. the proportion of actual below threshold instances that are incorrectly classified as above threshold), the area under the receiver operator curve (AUCROC) and the area under the precision-recall curve (AUCPR).

The dataset comprised of 21,768 ABR waves from 144 mice (Lab A = 48 mice; Lab B = 104 mice; Lab C = 36 mice), with each wave characterized by its frequency, decibel level, and amplitudes at 244 uniformly distributed sampling points over a 10 ms time window. As for the peak finding model, ABRs not initially sampled at 244 samples per 10 ms were truncated to 10 ms and either downsampled to 244 points with linear interpolation or upsampled to 244 points with cubic spline interpolation. The ABRs were grouped by subject and frequency, then 80% of these waveform stacks were randomly allocated for training and the remaining 20% were designated for testing (see Table 1 for ABR counts from each lab). This method ensures a representative distribution of ABRs from various subjects and frequencies across the training and testing sets. Accordingly, the training input matrix for the XGBoost classifier and the Logistic Regression Classifier had dimensions of 17,350 × 244, where 17,350 is the total number of training samples and 244 is the number of features, including 244 voltage readings for each ABR.

**Figure 4:**
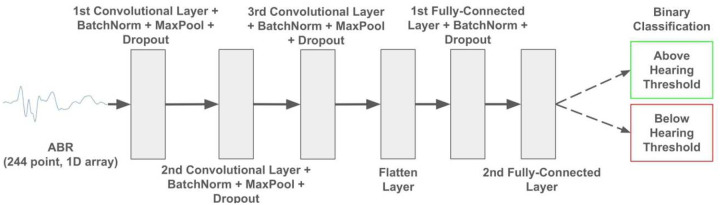
Model architecture for the CNN ABR threshold classifier. The input (a 244-point ABR waveform spanning 10 ms) passes through three sequential layers of convolution, batch normalization, max pooling (after ReLU activation), and dropout. The dimensionality of the output is reduced through two consecutive fully-connected layers using ReLU activation before finally being passed through a sigmoid activation function returning the classification of the ABR.

For the Logistic Regression Classifier and XGBoost Classifier, time warping was used on the ABR trajectories as an additional preprocessing step to align waveform features such as peaks and troughs (see Supplementary section: ABR Curve Alignment with Time Warping). ABRs were preprocessed as follows: a standardized scaler applied over the entire stack generated z-scores for each timepoint, and a min-max scaler on those z-scores scaled all values between 0 and 1. To improve generalization, data augmentation techniques were used to increase the sample space used for training. This included noise injection, elastic augmentation through cubic spline interpolation, and time shifting. Thus, the final training input matrix for the CNN had dimensions of 34,700 × 244, where 34,700 is the total number of training samples and 244 is the number of features, representing the 244 voltage readings for each ABR. Each training sample is weighted in the loss calculation to ensure that each lab’s data is represented equally in the model training. The architecture of the CNN is described in Figure 4.

## Results

### Peak Amplitude and Latency Estimation

To benchmark ABRA’s performance in peak amplitude and latency estimation, we fed a test set of 2,327 ABRs with human-labeled “ground truth” wave 1 amplitude and latency values from Lab A (80 waveforms from 8 mice) and Lab B (2,247 waveforms from 21 mice) into ABRA. The ground truth values for Lab A data were obtained by using visual examination with the BioSigRZ software from Tucker Davis Technologies, while the ground truth values for Lab B data were obtained using a semi-automatic approach using custom software (doi.org/10.5281/zenodo.14191510) (Carlton et al. 2024). Though it is possible to make manual adjustments to ABRA, we compare here only the differences resulting from the automated (i.e. unadjusted) estimates generated from ABRA vs their corresponding human-labeled ground truth values in order to fairly assess its underlying model.

**Figure 5:**
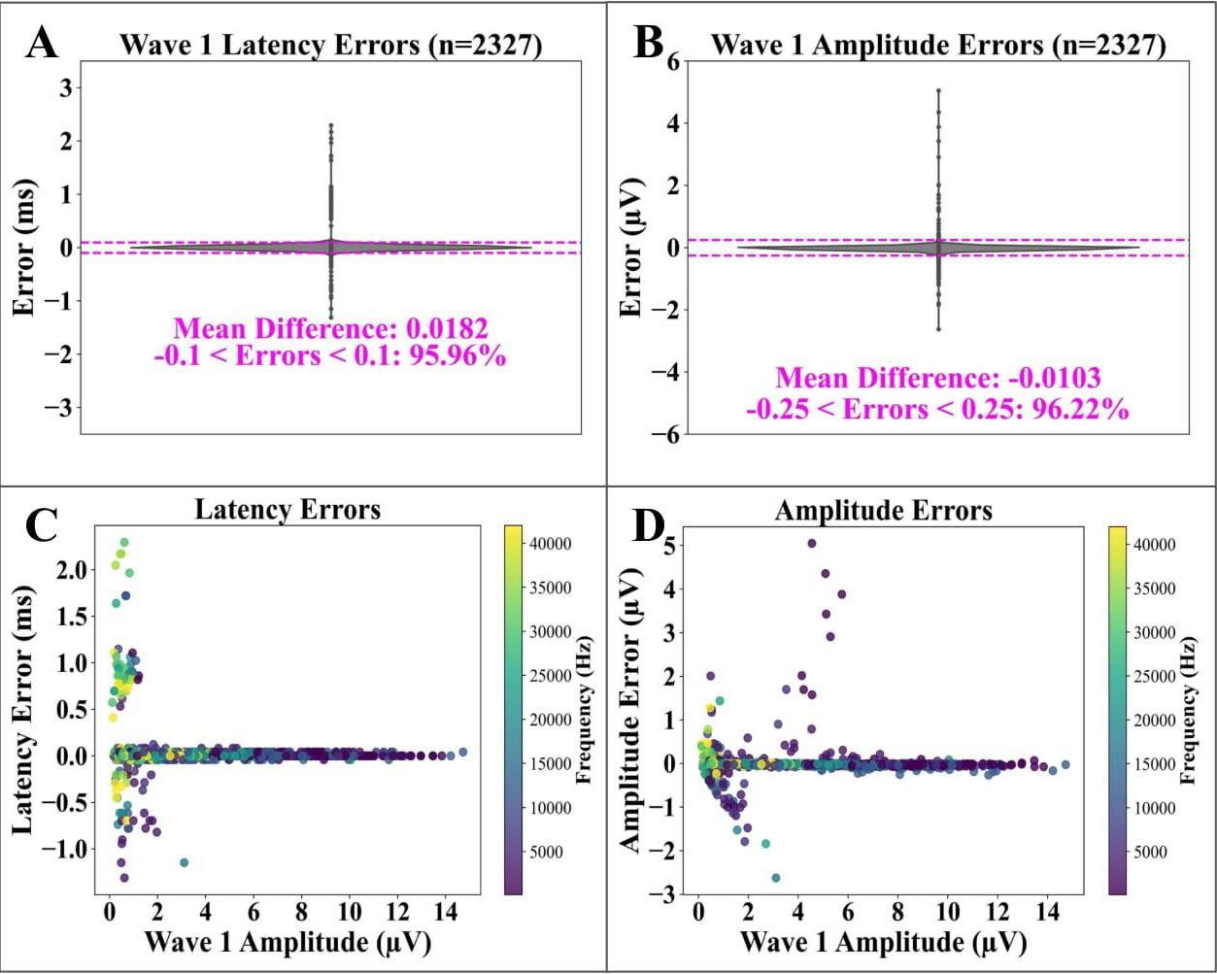
Errors in wave 1 peak quantification vs. ground truth for ABRA. (A) Errors in the predicted latency of the wave 1 peak (the direct output of the model). 95.96% of errors were within 0.1 ms of the ground truth measurement. (B) Errors in the wave 1 amplitude (peak to trough), calculated from the predicted wave 1 peak location, together with the estimated trough location. 96.22% of errors were within 0.25 μV of ground truth measurements. (C), (D). Wave 1 latency and amplitude errors vs. ground truth wave 1 amplitude, showing the highest errors occur on waveforms with small amplitudes (low SNR). n = 2327 represents the number of ABR waveforms in the test set. Related statistics are listed in Table 2.

Errors are defined as the differences between ABRA’s estimate and the ground truth value. Side-by-side swarmplots of the distributions of errors are shown for the latencies and amplitudes of the wave 1 peak in Figure 5A and 5B, respectively; summary statistics for errors are reported in Table 2. To further characterize error variability, we calculated the amplitude root mean squared error (*RMSE*_*a*_ = *0.2523*μV*, 95% CI: 0.2453–0.2598* μV), which indicates a relatively low level of variability in differences between ground truth amplitudes and ABRA estimates. The latency root mean squared errors (*RMSE*_*τ*_ = *0.1837 ms, 95% CI: 0.1786–0.1891* ms) suggest some variability in the range of differences between ground truth latency and ABRA estimates, though the mean absolute latency error (*MAE*_*τ*_ = *0.0415* ms) corresponds to only around 2.2% of the mean ground truth latency ($\bar{\tau }=1.8452\;\text{ms}$) or 0.4% of the total sweep length and is still indicative of consistent performance. These MAE intervals provide assurance that ABRA’s prediction errors for both amplitude and latency metrics are well bounded, but the ~4.5x higher RMSE values reflect occasional larger errors in rare instances.

**Figure 6:**
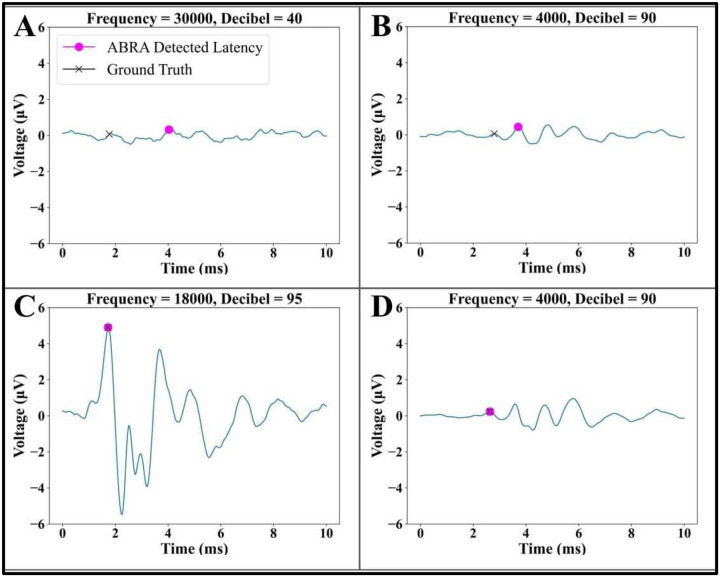
Examples of error and success cases in ABRA automated wave 1 peak detection. (A, B) For ABRs the hearing threshold and/or with low SNR, ABRA sometimes identified the incorrect peak. (C) and (D) display examples of ABR waveforms with varying signal to noise ratios for which ABRA matched the ground truth.

These comparisons show that ABRA-generated estimates generally agree with human-labeled ground truth latency and amplitude estimates. Figure 6 displays a few visual examples of how errors from the ABRA software may arise, with the most common source of errors arising from ABRs with very low signal-to-noise ratios (SNR).

### ABR Classification and Threshold Estimation Results

**Figure 7:**
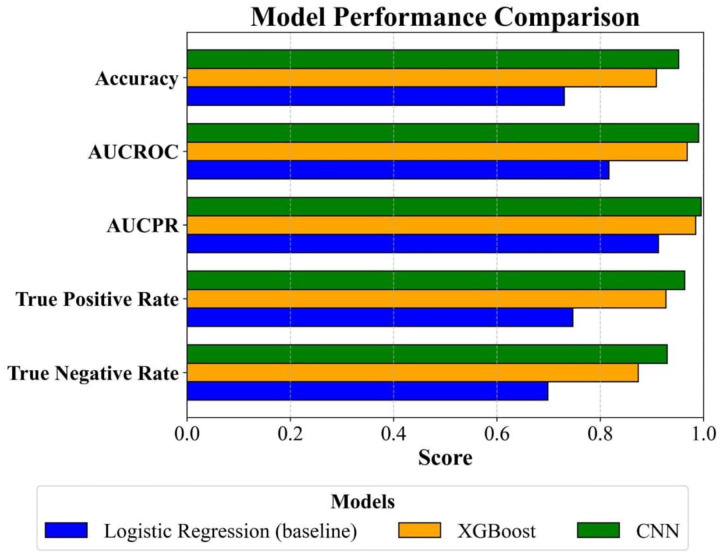
Comparative analysis of machine learning models for ABR threshold classification. Horizontal bar chart illustrating the performance of three machine learning models: Convolutional Neural Network (CNN), XGBoost, and Logistic Regression (baseline). The metrics used for comparison are Accuracy, Area Under the Receiver Operating Characteristic Curve (AUCROC), Area Under the Precision-Recall Curve (AUCPR), True Positive Rate, and False Positive Rate. Note that the false positive rate (FPR) is equal to the complement of the specificity, or true negative rate.

**Figure 8:**
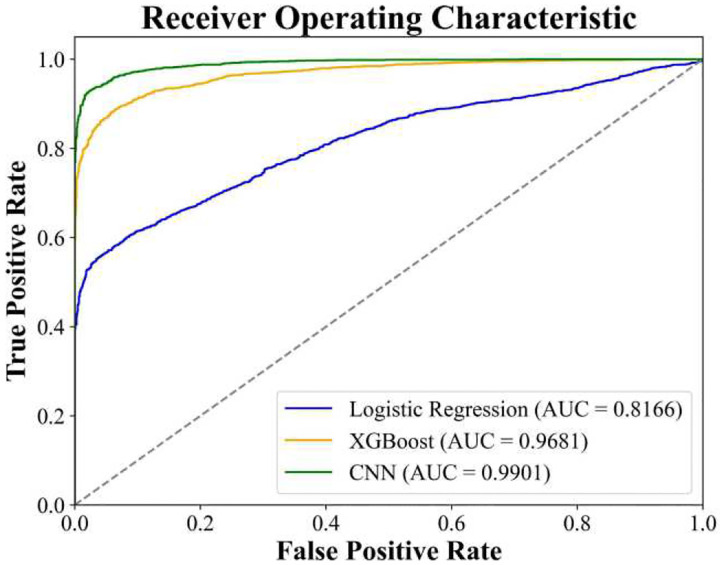
Receiver Operating Characteristic (ROC) curves for the three machine learning models for ABR threshold classification. The ROC curve demonstrates the performance of each ABR classifier (CNN, XGBoost, and Logistic Regression) at all classification thresholds. The area under the ROC curve (Area Under Curve; AUC) represents each ABR classifier’s overall ability to distinguish between above-threshold and below-threshold ABR responses. The ROC curve for the CNN shows slight outperformance of the XGBoost classifier, and both more significantly outperform the Logistic Regression classifier.

The performance of our ABR classifiers for threshold detection was assessed on the testing set of 4,490 ABR waveforms. Performance metrics are shown in Figure 7, and pairwise comparisons significance for these metrics is provided in Table 3. As a simple and interpretable model, logistic regression was used as a baseline for the binary classification task. It achieved an accuracy of 73.01%, a True Positive Rate (TPR), sometimes referred to as recall or sensitivity, of 74.68%, and an Area Under the Receiver Operating Characteristic Curve (AUCROC) of 0.8166. However, its performance was significantly outperformed by both the CNN and XGBoost models. The CNN surpassed XGBoost and the baseline Logistic Regression models in terms of all metrics, indicating the CNN’s enhanced ability to correctly identify ABR thresholds. The CNN model achieved both an AUCROC and an AUCPR of about 0.99 (Figure 7). These high values suggest strong overall discrimination at different decision thresholds (AUCROC, Figure 8) and excellent performance in handling class imbalance (AUCPR), reflecting robust sensitivity and precision.

**Figure 9:**
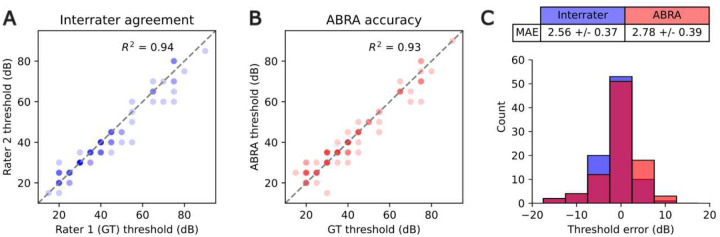
Threshold estimation accuracy of ABRA and agreement among multiple expert raters. (A,B) Comparison of threshold estimates between ground truth (GT) rater and a second expert (A) and between GT and the CNN-based ABRA method (B), across 90 measurements. Each threshold estimate is displayed as a semitransparent point such that darker points represent multiple overlapping values. (C) Distribution of threshold errors for the same 90 measurements, with interrater differences in blue and ABRA errors in red. The absolute errors were not statistically different between interrater and ABRA comparisons (p=0.68, t-test). The thresholds were estimated for 9 mice at 10 frequencies from Lab B. All data from Lab B was excluded from training and testing the CNN for this experiment.

The error in our method may partly stem from variations in the subjective determination of thresholds by different researchers, which were used as the ground truth for training our model. To quantify the variability between expert annotators, we sampled 90 ABRs (9 mice at 10 frequencies) from Lab B and asked an expert from Lab A to independently determine the thresholds. The difference between the thresholds assigned by Rater 1 (Lab B) and Rater 2 (Lab A) represents the interrater error. We then trained the CNN on only data from Labs A and C, so that data from Rater 1 was not included in training. On average, the interrater error was comparable to that of the CNN on the sample of Lab B data (Figure 9). While the mean absolute errors were similar, we also assessed the accuracy of the CNN predictions within 5 dB and 10 dB envelopes. At the 5 dB threshold, interrater accuracy exceeded that of the CNN by 1–3%, while at the 10 dB threshold, the two were indistinguishable (Table 4).

The CNN model’s superior performance at the 5dB and 10dB envelopes, along with its ability to handle the temporal nature of the data, makes it the optimal choice for this task. Moreover, the CNN achieves similar performance to the inter-rater comparison, indicating that its performance in estimating hearing thresholds is on par with the consensus of two human experts using standard methods. This suggests that the CNN model can function as a reliable tool for estimating hearing thresholds, providing a machine learning-based approach that matches human expert performance.

The performance of our threshold estimation technique was compared against that of EPL-ABR (Suthakar & Liberman 2019) on a separate dataset of ABR waveforms from Lab C (Table 5). This smaller set of ABR waveforms (N = 122) was selected because EPL-ABR’s threshold estimation software requires data in the custom ABR file format used by the Eaton Peabody Laboratories. Our CNN method outperforms EPL-ABR’s threshold estimation method across all metrics except for FPR on this dataset.

### Time Cost Analysis

In order to quantify the time savings of using ABRA, a random sample of ABR files from 10 mice at 9 frequencies each for a total of 90 waveform stacks from Lab B was analyzed by two ABR raters from Lab A. It took both raters approximately 1 hour to manually analyze the ABR thresholds. However, using ABRA, it took about 48 seconds to output the automated thresholds for all frequencies, corresponding to 75x increased efficiency. The automated thresholds were within 5 dBs of Lab A inspection 90% of the time, 10 dBs 98% of the time, and 15 dBs 100% of the time. For comparison, inter-rater assessment showed that a Lab A annotator was within 5 dB of a Lab B annotator’s result 92% of the time, 10 dB 98% of the time, and 15 dB 100% of the time.

## Discussion

The aim of this study is to illustrate ABRA’s versatility and additional benefits compared to other available software such as ABRWAS and EPL-ABR (see Table 6). ABRA has been designed to be a multi-purpose and versatile software with extended functionality to be able to handle datasets acquired from different mouse strains and experimental settings. It also facilitates processing of datasets recorded in different formats, including the widely used standard .arf files from BioSigRZ Tucker Davis Technology recordings, or a generalized.csv file format from any number of other systems. ABRA’s automated thresholding method also reduces the time required for thresholding analyses by more than 50x compared to manual analysis and can streamline the process of extracting ABR thresholds from multiple subjects. In addition, the results can be exported to a .csv file for post-processing by the experimenter, and plots can be directly exported for publication if desired.

The deep learning techniques used in ABRA have some precedence not only in previous ABR studies but in the field of electrophysiology in general. A recent study showed that convolutional neural networks and long short-term memory architecture can automatically detect discrete records of protein movement (Celik et. al. 2020). Another electrophysiological study introduced a deep learning algorithm that infers latent dynamics from single-trial neural spiking data (Pandarinath et. al. 2018). This study used data from non-overlapping recording sessions that improved the inference of their model, similar to how our software accounts for a range of data collection protocols for improved generalizability. Both studies were designed to automate otherwise laborious and arduous tasks and simplify them such that future studies can be more accurate, more reproducible, and less time-consuming. The deep learning techniques used in our software have similar potential for ABR studies by streamlining the onerous task of labeling peaks and identifying thresholds. We envision future ABR acquisition protocols that can be guided by our software to avoid acquiring excess measurements after a threshold is reached. Together, this work sets the stage for massively accelerated electrophysiology experimental and analytical workflows, powered by deep learning-based algorithms.

While we have argued that ABRA is a powerful tool for ABR analysis, it is necessary to remark that, like all existing ABR analysis programs, it also has several limitations that highlight areas for future development. Quality of life improvements for manual relabeling of ABRA-generated peaks, latencies, and thresholds for quality control are an area for future work. As for model limitations, the CNN-based thresholding model was trained only on mouse ABRs which had step sizes no larger than 20 dB; future work could extend or train new deep learning models to handle non-murine ABRs, which may have reduced signal-to-noise due to larger distances from the source generators. Validation of automated amplitude and latency measurements has so far been restricted primarily to wave 1, leaving waves 2–5 currently unvalidated; which can be pursued in future efforts as the model continues to incorporate new data from the labs mentioned here and others.

Another important consideration is the diversity of datasets used for model training: despite incorporating data from multiple laboratories and experimental conditions, ABRA’s models have yet to be extensively tested on highly diverse datasets, especially those involving severe mutations or disease conditions (e.g. noise-exposed, aged, or after exposure to ototoxic drugs) that could produce waveforms significantly different from the training data. Such conditions, representing “out-of-distribution” cases, may require retraining or fine-tuning of existing models. To address this challenge, transfer learning methods could be employed, rapidly adapting existing deep learning models to new data conditions with minimal additional training data. Furthermore, ABRA’s modular design allows its deep learning models to be used independently from the GUI, facilitating integration into other researchers’ computational workflows and software environments. Most importantly, the accuracy of peak and threshold detection may not yet match that of the most seasoned experts in visual ABR analysis for abnormal, ambiguous, or low signal-to-noise waveforms. While the time saved by automation may still yet be a worthwhile tradeoff for certain applications, an additional benefit is the deterministic nature of the model and therefore high reproducibility. Most importantly, we anticipate significant improvements in performance as larger and more diverse datasets are incorporated over time.

In summary, ABRA represents a significant advancement in auditory neuroscience, merging cutting-edge deep learning technology with accessible and intuitive software design. By automating the analysis of auditory brainstem responses—a crucial in vivo indicator of auditory function—ABRA not only accelerates hearing research but also enhances reproducibility and consistency across diverse experimental settings. Its user-friendly graphical interface, coupled with powerful computational models, makes advanced data processing accessible to researchers from various backgrounds, including neuroscientists, audiologists, and computational biologists. Beyond hearing research, ABRA exemplifies how artificial intelligence can effectively tackle complex biological data analysis, offering valuable insights into sensory neuroscience, neurodegenerative diseases, and beyond. Ultimately, ABRA provides a versatile and scalable solution, poised to facilitate transformative discoveries at the intersection of biology, medicine, and computer science.

## Supplementary Material

1

## Figures and Tables

**Table 1:** Breakdown of mouse and ABR waveform data used from the three different laboratories (Lab A, Lab B, Lab C) in the model split into a train and test datasets. Figures and Tables relevant to a given dataset are enumerated in brackets in the last row.

Lab/Model	Peak Detection		Automatic Thresholding		
	Training Data	Test Data	Training Data	Test Data	ABRA vs. EPL-ABR
**Lab A**	32 mice (288 ABRs)	8 mice (80 ABRs)	29 mice (5,335 ABRs)	9 mice (1,402 ABRs)	–
**Lab B**	83 mice (8,635 ABRs)	21 mice (2,247 ABRs)	75 mice (11,700 ABRs)	19 mice (2,966 ABRs)	–
**Lab C**	–	–	10 mice (243 ABRs)	2 mice (122 ABRs)	2 mice (122 ABRs)
**Total [Relevant Figures & Tables]**	**115 mice (8,923 ABRs)** [Fig. 3]	**29 mice (2,327 ABRs)** [Fig. 5, 6; Table 2]	**114 mice (17,278 ABRs)** [Fig. 4]	**30 mice (4,490 ABRs)** [Figs. 7–9; Tables 3, 4]	**2 mice (122 ABRs)** [Table 5]

**Table 2:** (related to Figure 5): Table showing the Mean Error Difference and their Standard Errors between ABRA-detected wave 1 latency and amplitude and corresponding ground truth values detected by human reviewers. Two sample t-tests found that mean error differences were not significant for wave 1 latency nor for wave 1 amplitude estimates at the 95% significance level after Bonferroni correction.

	Wave 1 Latency	Wave 1 Amplitude
Mean Difference (± S.E.M.)	ABRA vs. Ground Truth (ms)	ABRA vs. Ground Truth (μV)
Lab A (n_waveforms_=80, n_mice_=8)	0.0164 (±0.0306)	−0.0764 (±0.0491)
Lab B (n_waveforms_=2,247, n_mice_=21)	0.0183 (±0.0038)	−0.0080 (± 0.0051)
**Overall Test Set**	**0.0182 (±0.0038)**	−**0.0103 (±0.0052)**

**Table 3:** Comparative analysis of performance metrics between the three machine learning models for ABR threshold classification (related to Figure 8). Metrics were calculated on the test set of 4,490 ABR waveforms, and compared between the Convolutional Neural Network (CNN), XGBoost (XGB), and Logistic Regression (LR) models. The CNN model outperforms the XGB model across all metrics. Both CNN and XGB outperform the LR model across all metrics. All p-values were ~0 and statistically significant (at level 0.001) as calculated using either two-sample proportion tests (Accuracy, TPR, FPR) or bootstrapping (AUCROC, AUCPR), after applying a Bonferroni correction for multiple testing.

Metric	Comparison	Difference Estimate	95% CIs for differences
**Accuracy**	CNN vs. XGB	0.0432	(0.0430, 0.0434)
	CNN vs. LR	0.2216	(0.2213, 0.2219)
	XGB vs. LR	0.1784	(0.1780, 0.1788)
**AUCROC** Area Under the Receiver Operating Characteristic Curve	CNN vs. XGB	0.0221	(0.0221, 0.0221)
	CNN vs. LR	0.1736	(0.1736, 0.1736)
	XGB vs. LR	0.1515	(0.1515, 0.1515)
**AUCPR** (Area Under the Precision-Recall Curve)	CNN vs. XGB	0.0104	(0.0104, 0.0104)
	CNN vs. LR	0.0828	(0.0828, 0.0828)
	XGB vs. LR	0.0724	(0.0724, 0.0724)
**TPR** (True Positive Rate)	CNN vs. XGB	0.0364	(0.0360, 0.0367)
	CNN vs. LR	0.2165	(0.2160, 0.2170)
	XGB vs. LR	0.1801	(0.1796, 0.1807)
**TNR** (True Negative Rate)	CNN vs. XGB	0.0562	(0.0554, 0.0570)
	CNN vs. LR	0.2313	(0.2302, 0.2323)
	XGB vs. LR	0.1751	(0.1740, 0.1762)

**Table 4:** Inference for differences in accuracy between inter-rater accuracy (IR) and the Convolutional Neural Network (CNN) model in threshold estimation (related to Figure 9). Within a 10dB envelope, no significant difference between the CNN baseline and inter-rater accuracy was detected, suggesting CNN is performing at a comparable level as a human reviewer at this precision. At the more precise envelope of 5dB, there was a significant difference of 1–3% after Bonferroni correction.

Accuracy Envelope	Comparison	Accuracy Difference	95% CIs for Accuracy Difference	*p-value*	Significance
**Within 5dB**	CNN vs. IR	−0.0222	(−0.0310, −0.0134)	1.5578 × 10^−6^	***
**Within 10dB**	CNN vs. IR	0.0000	(−0.0046, 0.0046)	1.0	**NS**

Significance level notation after applying Bonferroni correction: 0.05 (*), 0.01 (**), 0.001(***).

**Table 5:** Performance comparison of threshold estimation algorithms on Lab C data only (122 ABR waveforms from 2 mice). This table presents a side-by-side comparison of two threshold estimation algorithms: EPL-ABR and ABRA. The metrics used for comparison include Accuracy, True Positive Rate (TPR), False Positive Rate (FPR), and the ability to estimate thresholds within 5dB, 10dB, and 15dB. The values are presented as mean (± standard error). ABRA demonstrates superior or equal performance (green cells/bolded) in terms of accuracy, TPR, FPR, and estimating thresholds within 5, 10, or 15dB.

Metric \ Software	EPL-ABR	ABRA
**Accuracy**	95.08 (±1.958)%	**96.72 (±1.612)%**
**True Positive Rate**	95.56 (±2.172)%	**97.78 (±1.554)%**
**False Positive Rate**	**6.25 (±4.279)%**	**6.25 (±4.279)%**
**Within 5dB**	91.67 (±7.979)%	**100.00 (±0.0)%**
**Within 10dB**	**100.00 (±0.0)%**	**100.00 (±0.0)%**
**Within 15dB**	**100.00 (±0.0)%**	**100.00 (±0.0)%**

**Table 6:** Comparison of software features/capabilities. Functionality and aspects of ABRA, the Auditory Brainstem Response Waveform Analyzer (ABRWAS) (Burke et al. 2023), and the EPL-ABR Peak Analysis App (Suthakar and Liberman 2019).

FEATURES	ABRA	ABRWAS	EPL-ABR
**Threshold Detection**	Automated thresholding with supervised machine learning methods	No automated threshold estimation	Automated thresholding using cross-covariance calculations
**Peak Detection**	Automates peak and trough detection	Suggests peak and trough detection as a guide for human revision	Automates peak and trough detection and allows for human revision
**Time Warping**	Performs elastic time warping	No time warping	No time warping
**Batch Processing**	Supports batch processing	Supports batch processing	Supports batch processing
**Data Extraction**	Generate peaks, troughs, and a metrics table with a single click	Generates peaks and troughs with option to manually adjust labels	Generates peaks and troughs with option to manually adjust labels using hotkeys
**Metric Exports**	Metrics table only includes three waveform metrics and the threshold for each frequency	Comprehensive metrics table	Comprehensive metrics table
**Accessibility**	Free and open source	Free and open source	Free and open source
**Image Exports**	Can export .png and .pdf files	No functionality	No functionality
**Stability**	When errors arise, app can recover easily	Errors require software relaunch	When errors arise, app can recover easily
**File Type Support**	Accepts .arfs and .csvs; only a couple rules related to the file structure	Each file must follow the same structure	Only supports EPL file type
**Operating System**	Windows/Mac/Linux	Windows	Windows/Mac
**Web Support**	Web-based application that can also run locally	Run on local machines only	Run on local machines only

## Data Availability

Data that support the findings of this study are publicly available from the following sources: Manor and Liberman Labs: https://zenodo.org/records/15626376; Marcotti Lab: https://zenodo.org/records/15619100. Scripts are publicly available in the Github: https://github.com/ucsdmanorlab/abranalysis.
