# Supplementary material for: An Open-Source Deep Learning-Based GUI Toolbox for Automated Auditory Brainstem Response Analyses (ABRA)
Source: Res Sq. 2025 Jun 20:rs.3.rs-6735294. Preprint. [Version 1] doi: 10.21203/rs.3.rs-6735294/v1 (PMC12204367; doi:10.21203/rs.3.rs-6735294/v1)
Supplement: 1 [file NIHPPRS6735294V1-supplement-1.pdf]

Supplementary Information

Getting Started with ABRA

A tutorial manual for ABRA can be found on the tool's Github (https://github.com/ucsdmanorlab/abranalysis) and at the following link.

Details on Data Collection

The three labs used a similar overarching methodology, including similar use of anesthesia, electrode placement, and decibel (dB) ranges, each used their own unique protocol, including varying collection software, sound source, and mouse strains. These differences are delineated in Table S1 and underscore the flexibility of ABRA in accommodating diverse experimental setups and protocols.

Methods	Lab A	Lab B	Lab C
**Anesthesia**	Ketamine (90 mg/kg) + Xylazine (10 mg/kg)	Ketamine (100 mg/kg) + Xylazine (10 mg/kg)	Ketamine (100 mg/kg) + Xylazine (10 mg/kg)
**Environment**	Soundproof chamber, heating pad (37°C)	Soundproof chamber, heating pad (37°C)	Soundproof chamber, heating pad (37°C)
**Electrode Placement**	Subcutaneous recording electrode at vertex, reference behind right pinna, ground on left leg	Subdermal electrodes behind pinna (reference and ground), vertex (active)	Needle electrodes: vertex to ipsilateral pinna (recording), ground near tail
**Sound Stimuli**	5-ms tone pips (0.5 ms cos2 rise-fall), 21/sec	5-ms pips (1.0-ms rise-fall with cos2 onset envelope), 42.6/sec	5-ms pips (0.5-ms rise-fall with cos2 onset envelope), 30/sec
**Recording**	Filtered (300 Hz - 3 kHz), averaged using BioSigRZ software, 512 responses averaged	Customized software (Ingham et al., 2011), RZ6 auditory processor, 256 responses averaged	Amplified (10,000X), filtered (100 Hz - 3 kHz), averaged with A-D board in LabVIEW system, 1024 responses averaged
**Probed frequencies**	4kHz, 8kHz, 16kHz, 24kHz, 32kHz	100 Hz, 3kHz, 6kHz, 12kHz, 18kHz, 24kHz, 30 kHz, 36kHz, 42 kHz	8kHz, 11.3kHz, 16kHz, 22.6kHz, 32kHz, 45.2kHz
**Sound Intensity**	Decreased from 90 dB to 10/20 dB in 5 dB steps	0-95 dB in 5 dB steps	Raised from ~10 dB below threshold to 80 dB in 5 dB steps
**Speaker distance**	Open-field - 10 cm from ear	Open-field - 10 cm from ear	Closed-field - ~3 cm from the eardrum
**Mouse age/strains used**	3-month SAMP8 (Senescence-Accelerated Mouse-Prone 8) (Takeda et al. 1981)	1-month C57Bl/6N with and without corrected CDH23	7-week C57Bl/6J

Supplementary Table S1: **Summary of the experimental recording conditions used by the three labs.** The specific methods employed by each lab—Manor Lab (Lab A), Marcotti Lab (Lab B), Liberman Lab (Lab C) in collecting each dataset are summarized, including anesthesia, environment, electrode placement, sound stimuli, response recording, sound frequencies and intensity, distance between the mouse and speaker, and mouse strains and ages.

Data that support the findings of this study are publicly available from the following sources: Manor and Liberman Labs: https://zenodo.org/records/15626376 ; Marcotti Lab: https://zenodo.org/records/15619100.

ABR Curve Alignment with Time Warping

ABRs from mice exhibit a characteristic structure with 5 distinct peaks (**Figure 1**). However, a common challenge in analyzing these ABR waveforms is the non-uniform latency across different frequencies and decibel levels. This variability in latency can distort functional summary statistics (e.g. mean ABR curve, covariance surface) and time-based comparisons of these responses, as the peaks do not occur at the same time across different ABRs for the same mouse. To address this, we provide an option to employ time warping to align these ABRs, which aligns the position of peaks and other salient features of the ABRs across time. This alignment decouples amplitude from latency variation, facilitating the visual comparison of amplitudes of ABR waveforms. The encoding of time alignment parameters into individual-specific warping functions provides the option of incorporating these features into machine learning models, which in some cases improves the models' performance and predictive power as it did for the Logistic Regression and XGBoost Classifiers for automated thresholding. Because time warping adjusts the spacing between points on the time axis (without affecting the amplitude), the time warped curves can be used to visually inspect threshold or peak amplitude, but not latency, which should be assessed on the original unwarped curves.

To conduct the time warping step, we use the *fdasrsf* package in Python (Tucker 2020). This package implements elastic time warping, a method that maximizes the alignment of key features in waveforms. Here, this technique provides aligned ABRs, counteracting the non-uniform latency across different frequencies and decibel levels.

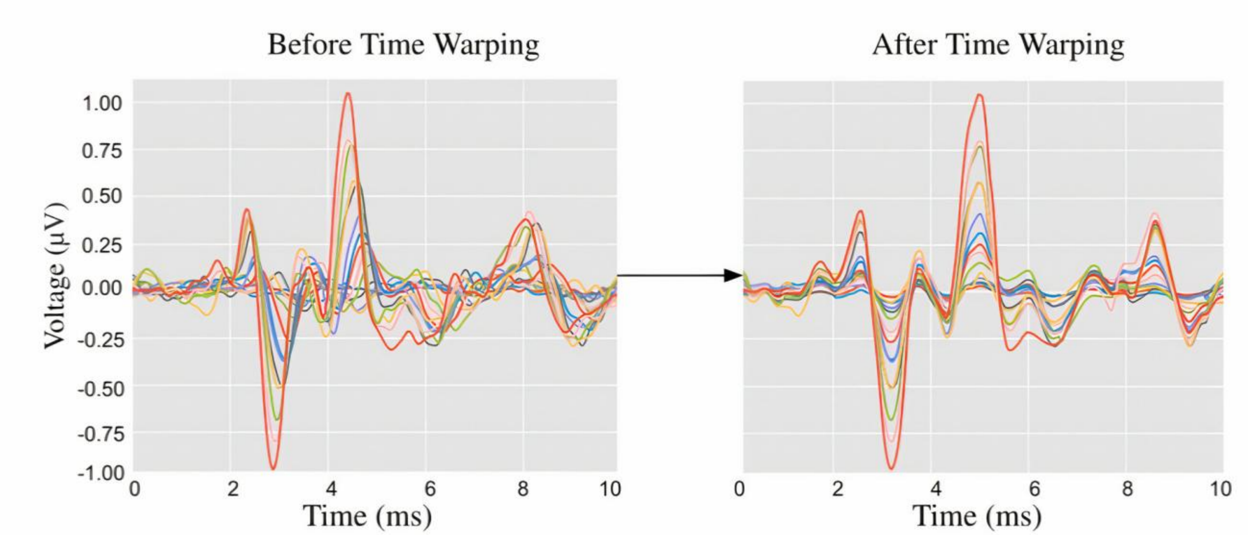

Supplementary Figure S1: ABRs before (left) and after (right) Time Warping. The depicted transformation of waveforms, both before and after applying elastic time warping using the *fdasrsf* package (Tucker 2020), illustrates clear registration of waveform features. Associated with each waveform is also an estimated time warping function which is useful in quantifying changes between the original unaligned latencies and the aligned latencies for all wave peaks and troughs.

ML Model Hyperparameters

Optimal choices of hyperparameters chosen by cross-validation for each of the thresholding and peak-finding models are displayed in Table S2 below.

	Thresholding CNN	Thresholding XGB	Thresholding LR	Peak Finding CNN
Hyper-parameters	Loss function: Binary Cross Entropy<br>Activation function for all layers except final layer: Relu<br>Final activation function: Sigmoid<br>Batch Size: 128<br>Early Stopping Patience: 25<br>Reduce Learning Rate on Plateau Patience: 20<br>Optimizer: Adam<br>Learning Rate: 1e-4<br>Conv. Layer 1 Filters: 128<br>Conv. Layer 2 Filters: 128<br>Conv. Layer 3 Filters: 64<br>Conv. Layer Stride: 1<br>Conv. Layer Padding: 0<br>Kernel Size: 7<br>MaxPool Size: 2<br>MaxPool Stride: 2<br>MaxPool Padding: 0<br>Fully Connected Layer Size: 128<br>Dropout Rate between Conv. Layers and before first Fully Connected Layer: 0.5<br>Dropout Rate between Fully Connected Layers: 0.4	Subsample size for each tree: 0.8<br>Positive class weight: 2<br>Boosting rounds: 700<br>Minimum sum of instance weight (hessian) in a leaf node: 1<br>Maximum depth of a tree: 5<br>Learning Rate: 0.05<br>Gamma (Regularization parameter for tree splitting): 0.5<br>Fraction of features used for fitting each tree: 1.0	All parameters are Scikit-learn defaults	Loss function: Mean Squared Error<br>Activation function for all layers: Relu<br>Batch Size: 32<br>Early Stopping Patience: 25<br>Optimizer: Adam<br>Learning Rate: 1e-3<br>Weight Decay: 1e-5<br>Conv. Layer 1 Filters: 128<br>Conv. Layer 2 Filters: 32<br>Conv. Layer Stride: 1<br>Conv. Layer Padding: 1<br>Kernel Size: 3<br>MaxPool Size: 2<br>MaxPool Stride: 2<br>MaxPool Padding: 0<br>Fully Connected Layer Size: 128<br>Dropout Rate between Conv. Layer 1 and Conv. Layer 2: 0.5<br>Dropout Rate between Conv. Layer 2 and first Fully Connected Layer: 0.3<br>Dropout Rate between Fully Connected Layers: 0.1
Library	Keras (3.3.3)	XGBoost (1.7.3)	Scikit-learn (1.2.2)	Pytorch (2.2.0.post100)

Supplementary Table S2: Candidate Model Hyperparameters for Thresholding and Peak Detection Models.
